# National and Regional Trends in Hemorrhagic Stroke Mortality in Brazil, 2000-2023

**DOI:** 10.7759/cureus.114600

**Published:** 2026-08-16

**Authors:** Jose I Silva Junior, Eduarda Lorrany do A Basaglia Santos, Pedro Petiti A Bueno, Chrystianne Ferreira da Silva B Cunha, Fernanda L Froes, Jessica N Tsurumaki, Ghiordana Milena D Lopes Guimarães, Vitor S Tellian Festa, Maria Eduarda D Prudente, Humberto G Moreira

**Affiliations:** 1 Faculty of Medicine, Universidade Federal Rural do Semi-Árido, Mossoró, BRA; 2 Faculty of Medicine, Anhembi Morumbi University, São Paulo, BRA; 3 Medical School, Universidade de Ribeirão Preto, Ribeirão Preto, BRA; 4 Faculty of Medicine, Centro Universitário Alfredo Nasser (UNIFAN), Goiânia, BRA; 5 Faculty of Medicine, Universidade Anhembi Morumbi, São Paulo, BRA; 6 Medicine, Universidade Nove de Julho, São Paulo, BRA; 7 Faculty of Medicine, Universidade de Rio Verde, Goiânia, BRA; 8 Faculty of Medicine, Universidade São Francisco, Bragança Paulista, BRA; 9 Medical School, Universidade Federal de Goiás, Goiânia, BRA

**Keywords:** brazil, health disparities, hemorrhagic stroke, mortality, time-series analysis

## Abstract

Introduction

Hemorrhagic stroke contributes disproportionately to stroke-related mortality and disability. This study examined national, regional, and sex-specific trends in hemorrhagic stroke mortality among Brazilian adults from 2000 to 2023 and assessed whether these trends changed during the COVID-19 pandemic.

Materials and methods

We conducted a population-based ecological time-series study using data from the Brazilian Mortality Information System. Deaths among adults aged 20 years or older were identified by International Classification of Diseases, 10th Revision codes I60-I62. Crude and age-standardized mortality rates were calculated per 100,000 population. Direct standardization used the age distribution of the 2022 Brazilian Census population. Weighted log-linear regression was used to estimate annual percentage changes (APCs) and 95% confidence intervals (CIs). Temporal slopes were compared by sex and geographic region. An interrupted time-series analysis with a fixed breakpoint at 2020 assessed immediate and postpandemic changes. Sensitivity analyses excluded 2020 and 2021 and compared crude with age-standardized rates.

Results

Between 2000 and 2023, 500,301 hemorrhagic stroke deaths were recorded. Annual deaths increased from 17,846 to 23,945, whereas the age-standardized mortality rate declined from 22.3 to 14.8 per 100,000 population. Mortality decreased nationally (APC, -2.09%; 95% CI, -2.36% to -1.82%) and among both men (APC, -1.98%; 95% CI, -2.25% to -1.71%) and women (APC, -2.18%; 95% CI, -2.47% to -1.90%). Regional trends differed: mortality was statistically stable in the North (APC, -0.08%; 95% CI, -0.51% to 0.35%) and Northeast (APC, -0.40%; 95% CI, -0.84% to 0.05%) and decreased in the Southeast, South, and Central-West. The interrupted time-series analysis found no significant immediate change in national mortality in 2020 (-6.3%; 95% CI, -15.6% to 4.2%). Excluding 2020 and 2021 did not materially change the direction or statistical classification of the national trend.

Conclusions

Age-standardized hemorrhagic stroke mortality declined in Brazil despite an increase in the absolute number of deaths, indicating that population growth and aging increased the mortality burden while age-specific risk decreased. National improvement concealed substantial regional disparities, with substantially less favorable trends in the North and Northeast than in the Southeast, South, and Central-West. These findings support prioritizing regional investigation of hypertension control, access to organized stroke care, and cause-of-death classification.

## Introduction

Stroke remains a leading cause of death and disability worldwide, with substantial variation in burden according to pathological subtype. Intracerebral hemorrhage accounted for 28.8% of incident strokes in 2021 but for 45.6% of stroke deaths and 49.5% of stroke-related disability-adjusted life-years. High systolic blood pressure remains the leading modifiable contributor to this burden [1]. Nontraumatic subarachnoid hemorrhage represents a distinct cerebrovascular condition with different causes, treatment pathways, and outcomes, and its mortality varies substantially across countries and sociodemographic settings [2].

Brazil has undergone rapid population aging while retaining marked regional differences in socioeconomic conditions, cardiovascular prevention, diagnostic capacity, and access to specialized stroke care. Previous national studies have reported declining cerebrovascular mortality overall, but broader stroke definitions may obscure differences in hemorrhagic subtypes and geographic trends. A national analysis of hospital admissions for spontaneous intracranial hemorrhage found high in-hospital mortality, increasing costs, limited use of neurosurgical procedures, and disruption during the COVID-19 pandemic [3]. Mortality surveillance is also affected by diagnostic specificity: unspecified stroke accounted for more than 60% of Brazilian deaths mentioning stroke in a recent analysis of individual death records [4]. Regional differences in stroke-unit access, treatment delays, and postacute rehabilitation may further contribute to variation in outcomes [5,6].

Long-term national trends in mortality specifically attributed to hemorrhagic stroke remain incompletely characterized in Brazil. Previous studies have generally focused on all cerebrovascular disease, hospital admissions, selected age groups, or shorter observation periods. The primary objective of this study was to characterize national, regional, and sex-specific trends in hemorrhagic stroke mortality among Brazilian adults from 2000 through 2023 using International Classification of Diseases, 10th Revision codes I60-I62. We also examined the divergence between absolute numbers of deaths and age-standardized mortality rates. Secondary analyses extended the observation period to 1996-2024 to assess the robustness of longer-term trends, while an exploratory segmented analysis evaluated temporal changes associated with the onset of the COVID-19 pandemic.

## Materials and methods

Study design and reporting

We conducted a population-based ecological time-series study of mortality from nontraumatic hemorrhagic stroke among adults aged 20 years or older in Brazil. The primary analysis covered January 1, 2000, through December 31, 2023. The extended series was constructed using the same case definition, age groups, mortality-data extraction, population matching, and age-standardization procedures as the primary 2000-2023 analysis, with the calendar window expanded to 1996-2024.

The study was reported in accordance with the REporting of studies Conducted using Observational Routinely-collected health Data (RECORD) statement and the Guidelines for Accurate and Transparent Health Estimates Reporting (GATHER) [7,8].

Data sources and study population

Mortality data were obtained from the Brazilian Mortality Information System (Sistema de Informações sobre Mortalidade), maintained by the Brazilian Ministry of Health and accessed through DATASUS. The system compiles information from death certificates registered throughout Brazil. Deaths were classified by year of occurrence, age, sex, and region of residence [9].

The study included deaths among people aged 20 years or older for which the underlying cause was classified according to the International Classification of Diseases, 10th Revision (ICD-10), as subarachnoid hemorrhage (I60), intracerebral hemorrhage (I61), or other nontraumatic intracranial hemorrhage (I62). These categories were combined to define nontraumatic hemorrhagic stroke. Analyses were conducted for Brazil overall and separately for men and women and for the five official geographic regions: North, Northeast, Southeast, South, and Central-West.

Annual resident-population denominators were obtained from the Brazilian Institute of Geography and Statistics (IBGE) and matched to mortality records by calendar year, age group, sex, and geographic region [10]. Deaths with unrecorded sex were retained in overall counts but excluded from sex-specific analyses. No missing values were imputed.

Mortality rates and age standardization

Crude mortality rates were calculated by dividing the annual number of deaths by the corresponding resident population and multiplying by 100,000. Age-specific mortality rates were calculated for seven age groups among adults aged 20 years or older: 20-29, 30-39, 40-49, 50-59, 60-69, 70-79, and ≥80 years.

Age-standardized mortality rates were calculated by direct standardization using the 2022 Brazilian population aged ≥20 years as the reference standard. Age-specific rates for the seven prespecified age groups were weighted according to their corresponding proportions in the 2022 standard population and summed. The age-specific standard-population counts and weights are provided in Appendix 1. For sex-specific analyses, standardization used the corresponding sex-specific 2022 age distribution; analyses of the overall population used the total 2022 age distribution. For each calendar year and population stratum, age-specific mortality rates were multiplied by the corresponding weights from the standard population and summed. All rates were expressed per 100,000 population. Crude rates were used to describe the observed mortality burden, whereas age-standardized rates were used for the primary temporal comparisons.

Statistical analysis

Annual numbers of deaths and crude and age-standardized mortality rates were summarized for Brazil overall and by sex and geographic region. The primary temporal analysis covered 2000 through 2023 and used weighted log-linear regression of the annual age-standardized mortality rate on calendar year. The dependent variable was the natural logarithm of the age-standardized rate, and observations were weighted by the corresponding annual number of deaths. Annual death counts were used as pragmatic precision weights because mortality rates based on larger numbers of events are generally estimated with greater precision. These weights were not intended to represent the inverse of the exact sampling variance of the age-standardized rates, and the model was not a Poisson generalized linear model.

Within each segment, annual percentage change (APC) was calculated as $100 \times [\exp(\beta_k) - 1]$, where β_k represents the corresponding log-linear slope. The residual variance from the weighted regression was used to estimate the standard error of β, and 95% confidence intervals (CIs) were calculated using the corresponding t distribution. Trends were classified as increasing when the APC and both confidence limits were greater than zero, decreasing when the APC and both confidence limits were less than zero, and stable when the 95% CI included zero.

Separate models were fitted for Brazil overall and for each sex and geographic region. Differences between independently estimated temporal slopes were assessed using z statistics calculated as $z = \frac{\beta_A - \beta_B}{\sqrt{SE(\beta_A)^2 + SE(\beta_B)^2}}$, where βA and βB represent the estimated log-linear temporal slope coefficients for the two groups being compared and SE(βA) and SE(βB) their corresponding standard errors. Two-sided p-values were derived from the standard normal distribution. These comparisons were exploratory, and no adjustment for multiple comparisons was applied.

Potential first-order serial correlation in the residuals of the primary national temporal model was assessed using the Durbin-Watson statistic.

Breakpoint and pandemic-period analyses

The association between the COVID-19 pandemic period and hemorrhagic stroke mortality was examined using segmented log-linear regression of the extended 1996-2024 series. A single breakpoint was specified at 2020. Specifically, the model was defined as $\ln(\mathrm{Rate}_t) = \beta_0 + \beta_1 D_t + \beta_2 t_{\mathrm{pre}} + \beta_3 t_{\mathrm{post}} + \varepsilon_t$, where Dₜ was an indicator equal to 1 for 2020 onward and 0 otherwise, tpre = year - 2020 before 2020 and 0 thereafter, and tpost = year - 2020 from 2020 onward and 0 previously. β₁ therefore estimated the immediate level change at 2020, whereas β₂ and β₃ represented the pre-2020 and post-2020 log-linear slopes, respectively. The breakpoint was selected on substantive grounds to represent the onset of the pandemic and was not estimated from the observed data. No joinpoint procedure or Bayesian information criterion-based breakpoint selection was used.

The immediate level change and the pre- and post-2020 slopes were transformed to percentage changes using the exponential transformation described above and reported with 95% CIs. Because only five annual observations were available from 2020 through 2024, estimates of the post-2020 slope were considered imprecise. Pandemic-period associations were interpreted as temporal associations and not as causal effects of COVID-19.

Sensitivity analyses

The robustness of the temporal findings was assessed in three ways. The extended 1996-2024 series was analyzed using unweighted log-linear regression to determine whether the principal findings were sensitive to the observation window and weighting approach. The trend models were repeated after excluding deaths recorded in 2020 and 2021 to assess whether the pandemic years disproportionately influenced the longer-term estimates. Trends in crude mortality rates were also compared with those in age-standardized rates to assess the extent to which changes in population age structure affected the observed patterns.

All tests were two-sided, and statistical significance was defined as a 95% CI that excluded the null value or a p-value of less than 0.05. The primary temporal models used the annual age-standardized mortality rate as the dependent variable. Statistical estimates generated in R were cross-checked against the analytical spreadsheet to verify consistency of regression coefficients, APCs, and corresponding confidence intervals. Analyses were performed using R version 4.6.1 (R Foundation for Statistical Computing, Vienna, Austria).

Ethical considerations

The study used aggregated, anonymized, publicly available data and involved no access to identifiable information. Ethics committee approval and informed consent were therefore not required under Brazilian regulations governing research using public-domain aggregated data.

## Results

Study population and mortality burden

**Figure 1:**
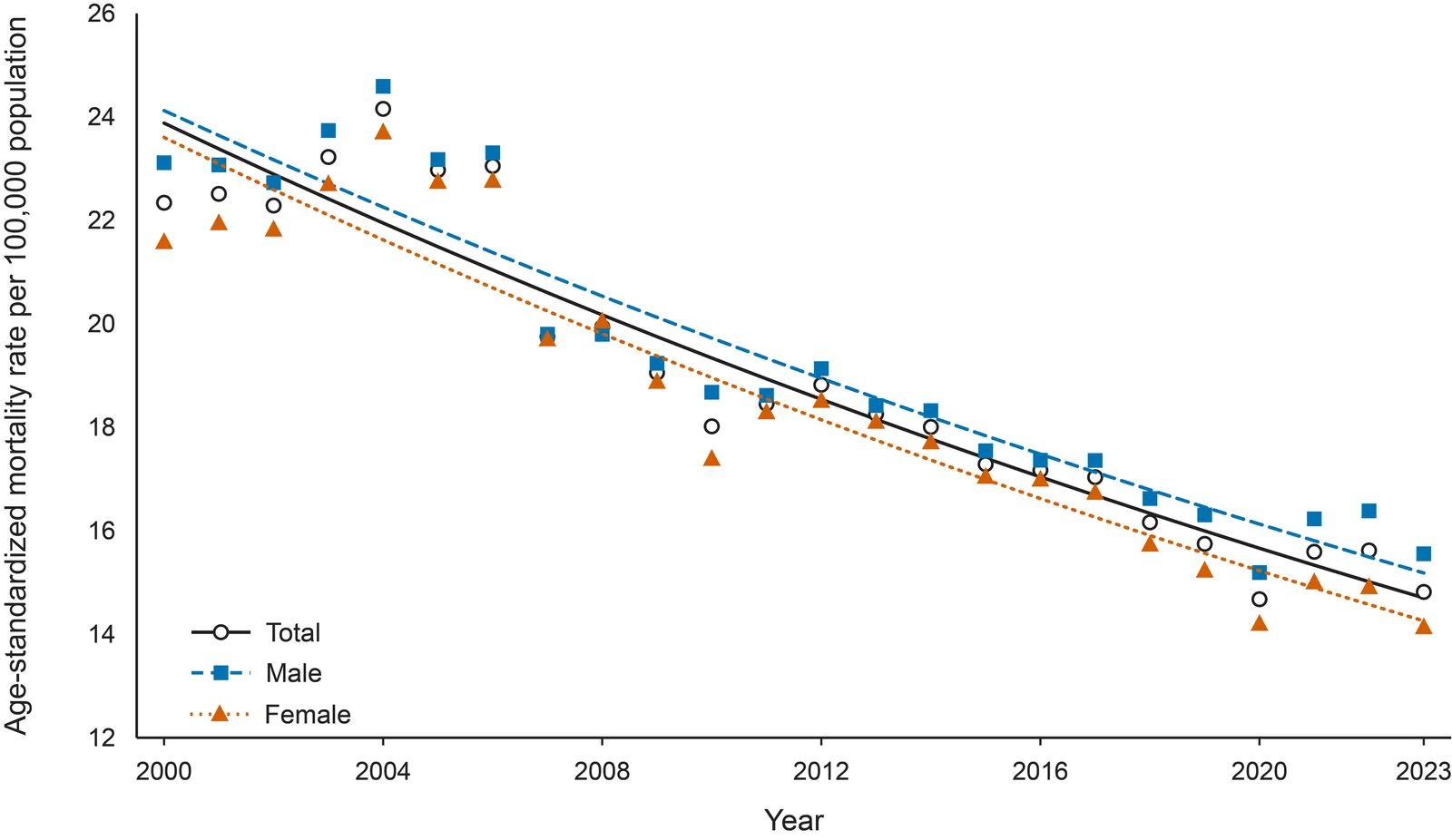
National trends in age-standardized mortality from nontraumatic hemorrhagic stroke by sex in Brazil, 2000-2023 Points represent annual mortality rates per 100,000 population, directly standardized to the age distribution of the 2022 Brazilian Census population. Lines represent exponential log-linear trends fitted over 2000-2023. Total mortality is shown by open black circles and a solid line; mortality among men by blue squares and a dashed line; and mortality among women by vermilion triangles and a dotted line. Deaths were defined using ICD-10 codes I60-I62. Source: Authors’ analysis of mortality data from the Brazilian Mortality Information System (SIM/DATASUS) [9] and population data from the Brazilian Institute of Geography and Statistics (IBGE) [10].

Between 2000 and 2023, 500,301 deaths from nontraumatic hemorrhagic stroke were recorded among adults aged 20 years or older in Brazil. Among deaths with recorded sex, 248,222 (49.6%) occurred in men and 252,010 (50.4%) in women. Sex was unrecorded for 69 deaths. The annual number of deaths increased from 17,846 in 2000 to 23,945 in 2023. Over the same period, the age-standardized mortality rate decreased from 22.3 to 14.8 per 100,000 population. Rates decreased from 23.1 to 15.6 per 100,000 among men and from 21.6 to 14.1 per 100,000 among women (Table 1 and Figure 1). Thus, the increase in the absolute number of deaths occurred alongside a reduction in age-standardized mortality.

**Table 1:** Deaths and mortality rates from non-traumatic hemorrhagic stroke (ICD-10 I60-I62), Brazil and regions, by sex, 2000 and 2023 *Crude mortality rate per 100,000 population. †Age-standardized mortality rate per 100,000 population (direct standardization; 2022 Brazilian Census population age structure as reference standard). Mortality data: Brazilian Mortality Information System (SIM/DATASUS), Ministry of Health. Population denominators: Brazilian Institute of Geography and Statistics (IBGE).

Region	Sex	Deaths, 2000 (n)	Deaths, 2023 (n)	Crude rate, 2000*	Crude rate, 2023*	Age-standardized rate, 2000†	Age-standardized rate, 2023†
Brazil	Total	17,846	23,945	17.3	15.1	22.3	14.8
	Male	8,997	12,068	18.0	15.8	23.1	15.6
	Female	8,847	11,877	16.6	14.4	21.6	14.1
North	Total	692	1,555	10.2	11.9	16.3	15.9
	Male	355	821	10.3	12.6	15.4	15.9
	Female	337	734	10.2	11.3	17.1	15.6
Northeast	Total	3,195	6,308	11.8	15.1	14.8	15.8
	Male	1,607	3,135	12.4	15.9	15.3	16.7
	Female	1,587	3,173	11.2	14.4	14.4	15.1
Southeast	Total	9,654	11,023	20.7	16.1	26.1	14.8
	Male	4,858	5,538	21.7	16.9	27.4	15.6
	Female	4,796	5,485	19.8	15.4	24.8	14.0
South	Total	3,073	3,271	19.3	14.1	24.9	12.6
	Male	1,527	1,669	19.8	14.9	25.6	13.3
	Female	1,545	1,602	18.8	13.3	24.3	12.0
Central-West	Total	1,232	1,788	17.5	14.4	26.9	15.9
	Male	650	905	18.7	15.0	27.4	16.0
	Female	582	883	16.3	13.9	26.0	15.7

National and sex-specific trends

In the primary 2000-2023 analysis, age-standardized mortality decreased nationally, with an APC of -2.09% (95% CI, -2.36% to -1.82%). Similar decreases were observed among men (APC, -1.98%; 95% CI, -2.25% to -1.71%) and women (APC, -2.18%; 95% CI, -2.47% to -1.90%).

There was no evidence that the temporal slopes differed between men and women nationally (z=0.47; p=0.64) or within the North (p=0.58), Northeast (p=0.28), Southeast (p=0.71), South (p=0.86), or Central-West (p=0.79). Mortality rates were higher among men at the beginning and end of the study period, but the proportional rates of decline were similar between sexes.

Regional trends

**Figure 2:**
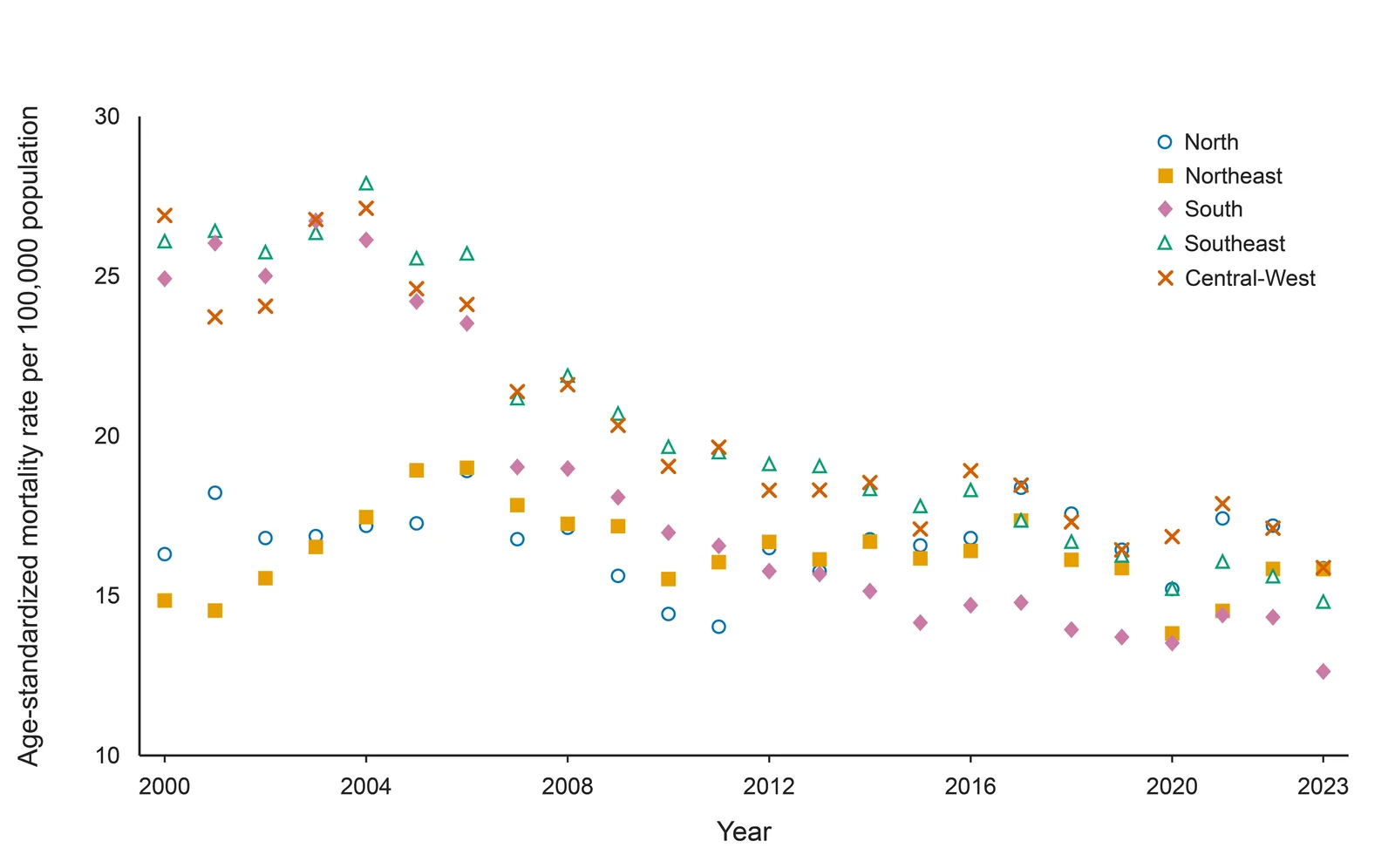
Age-standardized mortality from nontraumatic hemorrhagic stroke by geographic region in Brazil, 2000-2023 Points represent annual mortality rates per 100,000 population among adults aged 20 years or older, directly standardized to the age distribution of the 2022 Brazilian Census population. Regions are distinguished by color and marker shape: North, open blue circles; Northeast, gold squares; Southeast, open green triangles; South, purple diamonds; and Central-West, vermilion crosses. Deaths were defined using ICD-10 codes I60-I62. Source: Authors’ analysis of mortality data from the Brazilian Mortality Information System (SIM/DATASUS) [9] and population data from the Brazilian Institute of Geography and Statistics (IBGE) [10].

**Figure 3:**
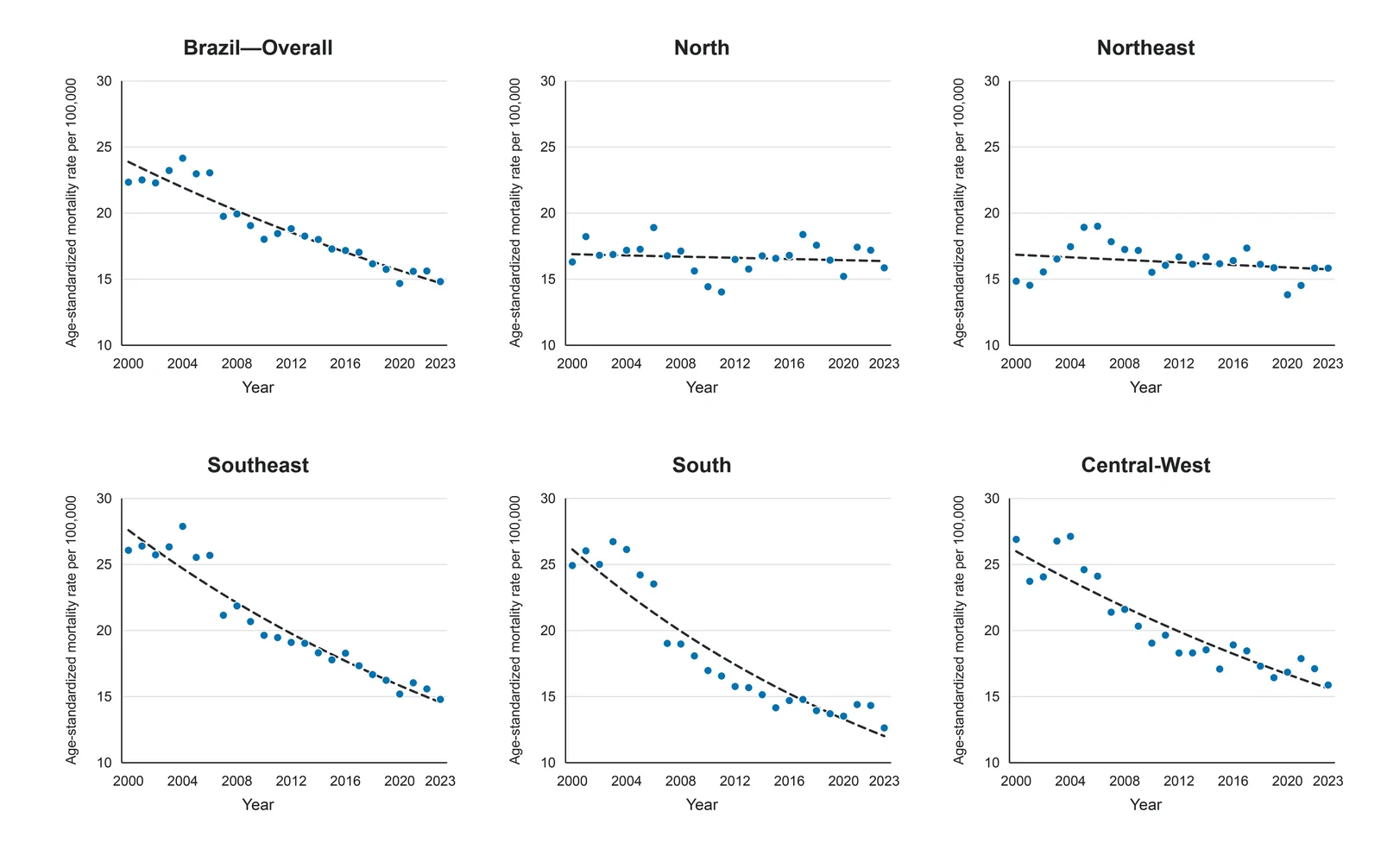
National and regional trends in age-standardized mortality from nontraumatic hemorrhagic stroke in Brazil, 2000-2023 Points represent annual mortality rates per 100,000 population, directly standardized to the age distribution of the 2022 Brazilian Census population. Dashed lines represent exponential log-linear trends fitted separately for Brazil and each geographic region over 2000-2023. All panels use the same y-axis scale. Deaths were defined using ICD-10 codes I60-I62. Source: Authors’ analysis of mortality data from the Brazilian Mortality Information System (SIM/DATASUS) [9] and population data from the Brazilian Institute of Geography and Statistics (IBGE) [10].

National estimates concealed substantial regional heterogeneity. From 2000 through 2023, age-standardized mortality was statistically stable in the North (APC, -0.08%; 95% CI, -0.51% to 0.35%) and Northeast (APC, -0.40%; 95% CI, -0.84% to 0.05%). Mortality decreased in the Southeast (APC, -2.74%; 95% CI, -3.03% to -2.45%), South (APC, -3.30%; 95% CI, -3.79% to -2.81%), and Central-West (APC, -2.14%; 95% CI, -2.52% to -1.76%) (Table 2; Figures 2-3).

**Table 2:** Temporal trends in age-standardized mortality rates from non-traumatic hemorrhagic stroke, Brazil and regions, by sex, 2000-2023 *Estimated from a weighted log-linear regression of the age-standardized mortality rate on calendar year, 2000-2023 (weights = annual deaths). Trend classified as increasing or decreasing when the 95% CI excluded zero, and stable otherwise. APC: annual percentage change; CI: confidence interval

Region	Sex	APC (95% CI), %/year*	Trend
Brazil	Total	-2.09 (-2.36 to -1.82)	Decreasing
	Male	-1.98 (-2.25 to -1.71)	Decreasing
	Female	-2.18 (-2.47 to -1.90)	Decreasing
North	Total	-0.08 (-0.51 to 0.35)	Stable
	Male	0.11 (-0.29 to 0.50)	Stable
	Female	-0.29 (-0.82 to 0.24)	Stable
Northeast	Total	-0.40 (-0.84 to 0.05)	Stable
	Male	-0.13 (-0.56 to 0.30)	Stable
	Female	-0.63 (-1.13 to -0.13)	Decreasing
Southeast	Total	-2.74 (-3.03 to -2.45)	Decreasing
	Male	-2.67 (-2.98 to -2.36)	Decreasing
	Female	-2.80 (-3.09 to -2.51)	Decreasing
South	Total	-3.30 (-3.79 to -2.81)	Decreasing
	Male	-3.31 (-3.84 to -2.77)	Decreasing
	Female	-3.29 (-3.76 to -2.83)	Decreasing
Central-West	Total	-2.14 (-2.52 to -1.76)	Decreasing
	Male	-2.06 (-2.50 to -1.62)	Decreasing
	Female	-2.20 (-2.56 to -1.83)	Decreasing

At baseline, the highest age-standardized mortality rates were observed in the Central-West (26.9 per 100,000), Southeast (26.1 per 100,000), and South (24.9 per 100,000). By 2023, the rates were 15.9 per 100,000 in the Central-West, North, and Northeast, compared with 14.8 in the Southeast and 12.6 in the South. The Northeast increased from the lowest regional rate in 2000 (14.8 per 100,000) to a rate comparable with those of the North and Central-West in 2023.

Pandemic-period analysis

In the exploratory segmented analysis of the extended 1996-2024 series, the estimated immediate change in national age-standardized mortality in 2020 was -6.3% (95% CI, -15.6% to 4.2%). The national trend before 2020 was decreasing (APC, -1.69%; 95% CI, -2.04% to -1.34%), whereas the post-2020 trend was statistically stable (APC, -0.08%; 95% CI, -3.80% to 3.77%).

Immediate changes were not statistically significant in most sex-specific and regional strata. An immediate decrease was observed in the Northeast overall (-20.1%; 95% CI, -31.9% to -6.2%), including men (-19.4%; 95% CI, -30.1% to -7.1%) and women (-20.8%; 95% CI, -34.1% to -4.7%). An immediate increase was observed among men in the South (14.3%; 95% CI, 0.7% to 29.8%). Post-2020 slopes were statistically stable in every national, regional, and sex-specific analysis. These estimates were imprecise because only five annual observations were available from 2020 through 2024.

Sensitivity analyses

In the extended 1996-2024 series, excluding 2020 and 2021 produced a national APC of -1.72% (95% CI, -2.00% to -1.45%), compared with -1.77% (95% CI, -2.03% to -1.52%) in the complete series. The direction and statistical classification remained unchanged nationally and in most regional and sex-specific analyses. In the Northeast overall, however, the trend changed from statistically stable in the complete extended series (APC, 0.31%; 95% CI, -0.15% to 0.78%) to increasing after exclusion of 2020 and 2021 (APC, 0.49%; 95% CI, 0.03% to 0.96%).

Analyses based on crude mortality rates showed smaller national declines than analyses based on age-standardized rates, indicating that population aging masked part of the reduction in mortality risk. This demographic effect did not account for the markedly less favorable age-standardized mortality trends observed in the North and Northeast compared with the other regions during the primary 2000-2023 analysis. Residual assessment of the primary national 2000-2023 model yielded a Durbin-Watson statistic of 1.01, indicating positive first-order serial correlation (Appendix 2).

## Discussion

From 2000 to 2023, the annual number of deaths from hemorrhagic stroke in Brazil increased from 17,846 to 23,945, while the age-standardized mortality rate decreased from 22.3 to 14.8 per 100,000 population. The decline was similar in men and women. National estimates, however, concealed substantial regional heterogeneity: mortality remained statistically stable in the North and Northeast while declining in the Southeast, South, and Central-West. The exploratory pandemic analysis found no immediate change nationally in 2020, and the limited number of post-2020 observations precluded firm conclusions about subsequent trends.

The increase in deaths despite declining age-standardized mortality is consistent with the contemporary global pattern for stroke, in which population growth and aging have increased the absolute burden while age-specific mortality has fallen [11]. Intracerebral hemorrhage continues to account for a disproportionate share of stroke deaths and disability [1], and subarachnoid hemorrhage remains an important source of premature mortality [2]. In the present analysis, crude mortality declined less than age-standardized mortality. Demographic change therefore obscured part of the national reduction in mortality risk, although the aggregate data do not separate the contributions of population growth and aging.

Regional divergence was the most consequential departure from the national trend. A Brazilian study restricted to hemorrhagic cerebrovascular disease reported increasing mortality in the North and Northeast between 2000 and 2019 [12]. By contrast, our primary 2000-2023 analysis found statistically stable age-standardized mortality in both regions, despite substantial declines elsewhere in Brazil. McBenedict et al. similarly reported declining average mortality across all macroregions, although the North and Northeast initially showed increasing rates followed by subsequent reductions [13]. Differences in case definition, standard population, follow-up period, and modeling approach may account for part of these discrepancies. Our extended 1996-2024 analysis likewise showed generally stable trends in the North and Northeast.

The mortality data cannot establish why regional trends differed. High systolic blood pressure is the leading modifiable contributor to hemorrhagic stroke burden globally and in Latin America [1,14]. Differences in hypertension detection and sustained treatment are plausible contributors, but neither was measured. Brazilian ecological evidence has also associated greater social vulnerability with less favorable trends in circulatory disease mortality [15]. That association supports further investigation of structural determinants; it does not show that socioeconomic conditions caused the regional patterns observed here.

Variation in stroke care provides another testable explanation. The SAMBA study documented differences in stroke lethality and functional outcomes among Brazilian cities, with the poorest outcomes in Sobral in the Northeast [16]. Implementation of organized stroke-unit care in a Brazilian hospital was associated with lower case fatality [5], while a Brazil-US study of aneurysmal subarachnoid hemorrhage reported longer treatment delays and worse outcomes in the Brazilian centers [6]. These studies assessed selected populations and cannot explain national mortality trends directly. They indicate that differences in service availability could influence survival and warrant linkage of mortality data with regional measures of stroke-care capacity. The pattern is also consistent with persistent international inequalities in stroke burden across sociodemographic settings [17].

Mortality rates were higher in men, but the slopes did not differ by sex nationally or within any region. This result indicates similar proportional changes over time; it does not imply equivalent mortality risk or exclude sex-specific differences in age distribution, risk factors, or care. Because the study was designed to compare temporal slopes, it cannot determine which mechanisms maintained the difference in rates between men and women.

No immediate national mortality change was identified at the fixed 2020 breakpoint. Hospital-based Brazilian studies reported changes in admissions, procedures, and resource use during the pandemic [18], but those outcomes are not directly comparable with population mortality. The Northeast showed an immediate decrease overall and in both sexes, whereas South men showed an increase; these subgroup findings were exploratory, arose from multiple comparisons, and require confirmation. Estimates after 2020 were imprecise because only five annual observations were available. The analyses therefore do not establish either the presence or absence of a sustained pandemic-related change.

The principal limitation is uncertainty in cause-of-death classification. Unspecified stroke accounts for a substantial proportion of Brazilian stroke records [4], and investigation of poorly specified cerebrovascular codes has reassigned some deaths to hemorrhagic stroke [19]. Restriction to I60-I62 increased specificity but probably reduced sensitivity, particularly where access to neuroimaging or certification quality was limited. Improvements in diagnostic specificity could also produce apparent changes over time or between regions. The ecological design precluded attribution of trends to hypertension control, socioeconomic conditions, or treatment. In addition, the primary trend estimates were derived from weighted log-linear regression of annual age-standardized rates, with annual death counts used as pragmatic precision weights rather than as inverse-variance weights. Residual assessment of the national model indicated positive first-order serial correlation (Durbin-Watson statistic, 1.01), suggesting that the conventional model-based standard errors and 95% CIs may be underestimated. Accordingly, the precision of the APC estimates should be interpreted with caution. Because the segmented pandemic-period model also did not explicitly incorporate a serial-correlation structure, similar caution applies to its conventional model-based standard errors, although residual autocorrelation was not separately quantified for that analysis. Regional pairwise comparisons and pandemic subgroup analyses were not designed as confirmatory causal tests. Finally, conclusions about the North and Northeast varied between the prespecified 2000-2023 period and the extended 1996-2024 series, underscoring the sensitivity of marginal trends to the analytical window.

## Conclusions

Age-standardized hemorrhagic stroke mortality declined nationally while the number of deaths increased, and the national decline was concentrated in the Southeast, South, and Central-West. Mortality trends in the North and Northeast were less favorable and depended partly on the period analyzed. Individual-level and health-system data are needed to determine whether regional differences reflect disease incidence, survival, cause-of-death classification, or a combination of these factors.

## Appendices

Appendix 1

**Table 3:** Age distribution and standardization weights of the 2022 Brazilian Census population used for direct age standardization Age-specific population counts and corresponding standardization weights for adults aged 20 years or older, derived from the 2022 Brazilian Census population. Separate weights are presented for the total population and for men and women because sex-specific mortality analyses used the corresponding sex-specific 2022 age distributions. Weights were calculated as the proportion of each age group within the relevant standard population and applied in the direct standardization of hemorrhagic stroke mortality rates.

Age group	Total population	Total weight	Male population	Male weight	Female population	Female weight
20-29	33,819,144	0.21740	17,038,567	0.22807	16,780,577	0.20754
30-39	34,206,034	0.21989	16,905,495	0.22629	17,300,539	0.21397
40-49	30,488,714	0.19599	14,765,027	0.19764	15,723,687	0.19447
50-59	24,554,480	0.15784	11,680,423	0.15635	12,874,057	0.15923
60-69	17,860,444	0.11481	8,207,071	0.10986	9,653,373	0.11939
70-79	9,835,161	0.06322	4,289,138	0.05741	5,546,023	0.06859
≥80	4,798,160	0.03084	1,822,200	0.02439	2,975,960	0.03681
Total	155,562,137	1.00000	74,707,921	1.00000	80,854,216	1.00000

Appendix 2

**Table 4:** Durbin-Watson residual autocorrelation diagnostic for the primary national weighted log-linear model, Brazil, 2000-2023 The Durbin-Watson statistic was calculated from the residuals of the primary weighted log-linear regression of annual age-standardized mortality rates from nontraumatic hemorrhagic stroke on calendar year for Brazil overall, 2000-2023, with annual death counts used as pragmatic precision weights. A Durbin-Watson statistic of 2 is consistent with absence of first-order serial correlation, whereas values below 2 are consistent with positive serial correlation. The observed statistic was 1.01, indicating positive first-order residual serial correlation.

Year	ln(Rate) actual	Predicted ln(Rate)	Residual e	e(t) - e(t-1)	[e(t)-e(t-1)]²	e(t)²
2000	3.10638007	3.173963351	-0.067583281			0.0045675
2001	3.114050859	3.152857171	-0.038806312	0.028776969	0.000828114	0.00150593
2002	3.103954503	3.131750991	-0.027796488	0.011009824	0.000121216	0.000772645
2003	3.145186493	3.11064481	0.034541683	0.06233817	0.003886047	0.001193128
2004	3.184464746	3.08953863	0.094926116	0.060384433	0.00364628	0.009010967
2005	3.134298574	3.06843245	0.065866124	-0.029059992	0.000844483	0.004338346
2006	3.137703128	3.04732627	0.090376858	0.024510734	0.000600776	0.008167976
2007	2.983333633	3.02622009	-0.042886457	-0.133263315	0.017759111	0.001839248
2008	2.992618636	3.00511391	-0.012495273	0.030391184	0.000923624	0.000156132
2009	2.947356977	2.984007729	-0.036650753	-0.02415548	0.000583487	0.001343278
2010	2.891416138	2.962901549	-0.071485411	-0.034834658	0.001213453	0.005110164
2011	2.915276768	2.941795369	-0.026518601	0.044966809	0.002022014	0.000703236
2012	2.935021918	2.920689189	0.014332729	0.04085133	0.001668831	0.000205427
2013	2.904581925	2.899583009	0.004998916	-0.009333813	8.71201 × 10⁻⁵	2.49892 × 10⁻⁵
2014	2.890696604	2.878476829	0.012219775	0.007220859	5.21408 × 10⁻⁵	0.000149323
2015	2.849957711	2.857370648	-0.007412938	-0.019632713	0.000385443	5.49516 × 10⁻⁵
2016	2.843231517	2.836264468	0.006967049	0.014379987	0.000206784	4.85398 × 10⁻⁵
2017	2.835320572	2.815158288	0.020162284	0.013195235	0.000174114	0.000406518
2018	2.782714879	2.794052108	-0.011337229	-0.031499513	0.000992219	0.000128533
2019	2.756667834	2.772945928	-0.016278094	-0.004940865	2.44121 × 10⁻⁵	0.000264976
2020	2.686294782	2.751839748	-0.065544966	-0.049266872	0.002427225	0.004296143
2021	2.746853463	2.730733567	0.016119895	0.081664861	0.00666915	0.000259851
2022	2.748560137	2.709627387	0.038932749	0.022812854	0.000520426	0.001515759
2023	2.695831097	2.688521207	0.00730989	-0.03162286	0.001000005	5.34345 × 10⁻⁵
